# A Genome-Wide Association Study of Attention Function in a Population-Based Sample of Children

**DOI:** 10.1371/journal.pone.0163048

**Published:** 2016-09-22

**Authors:** Silvia Alemany, Natàlia Vilor-Tejedor, Mariona Bustamante, Jesús Pujol, Dídac Macià, Gerard Martínez-Vilavella, Raquel Fenoll, Mar Alvárez-Pedrerol, Joan Forns, Jordi Júlvez, Elisabet Suades-González, Sabrina Llop, Marisa Rebagliato, Jordi Sunyer

**Affiliations:** 1 ISGlobal, Centre for Research in Environmental Epidemiology (CREAL), Barcelona, Spain; 2 Universitat Pompeu Fabra (UPF), Barcelona, Spain; 3 CIBER Epidemiology and Public Health (CIBERESP), Barcelona, Spain; 4 Centre for Genomic Regulation (CRG), The Barcelona Institute of Science and Technology, Barcelona, Spain; 5 MRI Research Unit, Department of Radiology, Hospital del Mar, Barcelona, Spain; 6 Centro Investigación Biomédica en Red de Salud Mental, CIBERSAM G21, Barcelona, Spain; 7 Department of Genes and Environment, Division of Epidemiology, Norwegian Institute of Public Health, Oslo, Norway; 8 Learning Disabilities Unit (UTAE); Neuropediatrics Department, Hospital de Sant Joan de Déu, Universitat de Barcelona, Barcelona, Spain; 9 Epidemiology and Environmental Health Joint Research Unit, FISABIO−Universitat Jaume I−Universitat de València, Valencia, Spain; 10 University Jaime I (UJI), Castellón, Spain; 11 IMIM (Hospital del Mar Medical Research Institute), Barcelona, Spain; Kunming Institute of Zoology, Chinese Academy of Sciences, CHINA

## Abstract

**Background:**

Attention function filters and selects behaviorally relevant information. This capacity is impaired in some psychiatric disorders and has been proposed as an endophenotype for Attention-Deficit/Hyperactivity Disorder; however, its genetic basis remains largely unknown. This study aimed to identify single nucleotide polymorphism (SNPs) associated with attention function.

**Materials and Methods:**

The discovery sample included 1655 children (7–12 years) and the replication sample included 546 children (5–8 years). Five attention outcomes were assessed using the computerized Attentional Network Test (ANT): alerting, orienting, executive attention, Hit Reaction time (HRT) and the standard error of HRT (HRTSE). A Genome-wide Association Study was conducted for each outcome. Gene set enrichment analyses were performed to detect biological pathways associated with attention outcomes. Additional neuroimaging analyses were conducted to test neural effects of detected SNPs of interest.

**Results:**

Thirteen loci showed suggestive evidence of association with attention function (P<10^−5^) in the discovery sample. One of them, the rs4321351 located in the *PID1* gene, was nominally significant in the replication sample although it did not survive multiple testing correction. Neuroimaging analysis revealed a significant association between this SNP and brain structure and function involving the frontal-basal ganglia circuits. The mTOR signaling and Alzheimer disease-amyloid secretase pathways were significantly enriched for alerting, orienting and HRT respectively (FDR<5%).

**Conclusion:**

These results suggest for the first time the involvement of the *PID1* gene, mTOR signaling and Alzheimer disease-amyloid secretase pathways, in attention function during childhood. These genes and pathways have been proposed to play a role in neuronal plasticity, memory and neurodegenerative disease.

## Introduction

Attention is a cognitive function essential in daily life. Every day, our perceptual systems are exposed to a massive internal and external sensory input for which the relevant behavioural information is selected and prioritized [1, 2]. Attention function allows the selection and prioritization of stimuli by activating different neural systems that interact with each other in a complex manner. It has been proposed that three functionally and anatomically different networks are involved in this process: alerting, orienting and executive attention [3]. According to Posner and Rothbart (2009), ‘alerting is defined as achieving and maintaining a state of high sensitivity to incoming stimuli; orienting is the selection of information from sensory input; and executive attention involves mechanisms for monitoring and resolving conflict among thoughts, feelings, and responses’ [3].

From a developmental perspective, attention constitutes a precursor of other higher-level cognitive abilities such as learning and memory [4]. Therefore, attention function plays a key role in cognitive development. From a clinical perspective, attention function is impaired in neuropsychiatric disorders such as Attention-Deficit/Hyperactivity Disorder (ADHD) and schizophrenia [5–9], thus, research in attention may have etiological implications for these disorders. These facts highlight the relevance of investigating the sources of variation of attention function.

Although experiences with the caregivers, education and other exposures account for individual variation in cognitive functions, the development of attention is partly specified by genes [3]. Genetic effects on attention function variation have been found early in life [10]. The catechol-O-methyltransferase gene (*COMT*) and the dopamine D4 receptor gene *(DRD4*) are among the candidate genes for attention function development [1, 10, 11]. However, these studies found modest associations and they failed to identify consistent and replicable results. A twin study including only 26 pairs estimated the heritability of the abovementioned attention networks ranging from 0% for orienting to 72% for executive attention [12]. Other studies report low to moderate (28–38%) and high (79%) heritability estimates for attention function [13, 14]. Therefore, although we can expect genetic influences on attention function development, there is still scarce knowledge about the genetic basis of attention in general population. Furthermore, to our knowledge, there are no previous genome-wide association studies (GWAS) on attention function during childhood.

The main goal of the present study was to identify common genetic variants associated with attention at a genome-wide level. The steps followed include: i) identification of single nucleotide polymorphisms (SNPs) associated with attention, ii) replication of significant findings in an independent sample, iii) search for relevant biological pathways accumulating associated genetic variants using gene set enrichment analyses (GSEA) and iv) examination of potential association between relevant identified SNPs and variations in brain structure and function using neuroimaging tools.

## Materials and Methods

### Discovery sample

The discovery sample was obtained from the BRain dEvelopment and Air polluTion ultrafine particles in scHool childrEn (BREATHE) project aimed to analyze the association between air pollution and cognitive development of scholars [15]. From the total of 2897 children participating in this project, genotypic and neurocognitive data was available for 1655 individuals. All parents and legal guardians signed the informed consent approved by the Clinical Research Ethical Committee (No. 2010/41221/I) of the Institut Hospital del Mar d’Investigacions Mèdiques–Parc de Salut Mar, Barcelona, Spain.

### Replication sample

The replication sample included 546 children from the INfancia y Medio Ambiente (INMA) multicenter birth cohort project recruited in the cities of Sabadell and Valencia (Spain) (INMA-SabVal) [16]. All parents and legal guardians signed the informed consent approved by the Clinical Research Ethical Committee of the Institut Hospital del Mar d’Investigacions Mèdiques–Parc de Salut Mar, Barcelona and institutional ethics committees in each region.

### Measures

Attention function was assessed using the computerized Attentional Network Test [ANT; [17]] which assesses three attentional networks: alerting, orienting and executive attention. The computerized version of this test has been validated with brain imaging [17] and in the general population [18]. As part of the BREATHE project, a follow-up with four repeated measurements of the attention function were conducted in a period of a year. The outcomes analyzed herein correspond to the first administration of the test. Five attention outcomes were analyzed in the current study: alerting, orienting, executive attention, hit-reaction time (HRT) and the standard error of the HRT (HRTSE). Reaction time (RT) measures (i.e. time between the introduction of a stimulus and the reaction on the subject to that stimulus) were used to calculate alerting (RT for no cue minus RT for double cue trials), orienting (RT for central cue minus RT for spatial cue trials) and executive function (RT for incongruent minus RT for congruent trials) scores. HRT (Median RT for correct responses) and HRTSE (Standard error or RT for correct responses) were also analyzed as measures of variability. All the outcomes analyzed were continuous variables. Higher scores indicate worse performance. Children with >30% errors were excluded from the analysis. Further details can be found elsewhere [18].

### Study Design, Genotyping and quality control

To identify novel loci associated with attention outcomes, we conducted a GWAS with follow-up of associations at suggestive evidence (P<10^−5^) in the replication sample.

In the discovery sample, DNA samples from 2492 children were obtained from saliva collected in Oragene DNA OG-500kit (DNA Genotek) following instructions of the manufacturer with minor modifications. DNA samples were quantified using Quant-iT^™^ PicoGreen^®^ dsDNA Assay Kit (Life Technologies). A final subset of 1778 children was selected for genome-wide genotyping after applying a filtering criteria (low quality DNA, no neuropsychological data, non Caucasian descent origin and not born in Spain, parents born in Europe, and adopted children). Genome-wide genotyping for the discovery sample was performed using the HumanCore BeadChip WG-330-1101 (Illumina) at the Spanish National Genotyping Centre (CEGEN) coordinated by the Spanish National Cancer Research Centre CNIO). Genotype calling was done using the GeneTrain2.0 algorithm (with a default threshold of 0.15) based on HapMap clusters implemented in the GenomeStudio software. Twenty CEU HapMap duplicates and twenty BREATHE duplicates were included in the study and gave consistent results.

PLINK was used for the genotyping quality of the sample and SNPs [19]. Quality control procedures were samples with a minimum of 97% call rate (N = 3 exclusions) and a maximum of 4 SD heterozigosity were included (N = 5 exclusions). Further checking was conducted for gender discordance excluding mismatch information (N = 18 exclusions, representing 1% of the sample), sample relatedness excluding proportions of identity by-state above 0.185 (N = 80 exclusions: 1 twin, 32 siblings, 39 cousins, 8 incongruent sibling's couples) and population stratification. Five subjects were excluded due to mental disabilities. In total we excluded 111 subjects (6.26%) leaving 1667 individuals from who 1655 have data available for the attention outcomes considered in the present study.

Genetic variants were filtered by Hardy-Weinberg equilibrium (P<10^−6)^, allele frequency (excluding minor allele frequency (MAF<1%) and SNP call rate with a minimum of 95%. In total, 58827 genetic variants (19.68%) were excluded. The final discovery genetic data set included 240103 SNPs.

The replication cohort was genotyped using the HumanOmni1-Quad v1.0 Beadchip (Illumina) at the CEGEN. Quality control procedures for the replication sample were also performed in PLINK. Samples with a minimum of 98% call rate and a maximum of 3 SD heterozigosity were included. Furthermore, gender discordance, sample relatedness (excluding proportions of identity by-state above 0.185) and population stratification were checked. Genetic variants were filtered using the same criteria as in the discovery sample. The final replication sample included 546 subjects.

### Statistical Analysis

We used a two-sample t-test to check for differences in ANT outcomes and age, and a Pearson’s χ^2^ test to check for sex differences. Genome-wide association analyses were conducted using linear regression models in SNPtest [20]. Separate models were tested for each ANT outcome as dependent variables and genetic markers as predictors. Additive genetic models were assumed to assess the association of each SNP with each ANT outcome, adjusting for age, sex and school.

Quantile-quantile (Q-Q) and Manhattan plots were computed using the qqman package of R. Genome-wide significance was set at P<5x10^-8^, and suggestive evidence of association was set at P<10^−5^. These thresholds have been recommended by a simulation study taking into account linkage disequilibrium (LD) between SNPs [21]. SNPs showing an association with attention outcomes (at GWAS or suggestive significance) were taken forward for replication in the INMA-SabVal sample. In the replication sample, multiple linear regressions in SNPtest adjusting by age, sex and cohort were conducted. In order to be replicated, SNPs must be nominally significant (P<0.05) after multiple testing correction (FDR<0.05).

To further analyze the association signal, regions which include SNPs of potential interest for cognition were imputed using IMPUTE2 v2 [20] taking the 1000 Genomes project phase I integrated variant set (http:/www.1000genomes.org/) as a reference haplotype panel. Regional association plots were computed with LocusZoom [22].

In addition, potentially relevant SNPs detected were analyzed for associations with gene expression using the Brain expression quantitative trait loci (eQTL) Almanac (http://www.braineac.org/) [23]. BRAINEAC is a publicly accessible database which contains gene expression data (generated eQTL) analyzed in ten brain regions from postmortem human brains.

Gene set enrichment analyses (GSEA) were conducted using Meta-Analysis Gene-set Enrichment of variaNT Associations (MAGENTA) software (19) for each attention outcome. Data sources included Reactome, Panther, KEGG and Ingenuity. As described in detail previously [24], MAGENTA individually mapped genes in the genome to the lowest P-value single SNP within a 110kb upstream and 40kb downstream window. These P-values were adjusted for confounding factors (e.g. physical gene size, number of SNPs per kilobase for each gene and other genetic properties). Genes are then ranked according to these adjusted P-values, and the gene-set enrichment P-value for each biological pathway was calculated for a given significance threshold (95^th^ percentile). To test whether genes were enriched in a pathway more than would be expected by chance, this value was compared with that generated with randomly permuted pathways of identical size. Individual pathways that reached FDR<0.05 were deemed significant and results for the 95^th^ percentile cut-off analysis were reported.

### Neuroimaging analyses

To further understand the role of SNPs of potential interest for cognition, its effects on brain structure and function were examined in a subsample of 185 children drawn from the BREATHE project who underwent neuroimaging studies with genetic and cognitive data available. More details in [25]. The imaging approach included whole-brain mapping of cortical thickness using high resolution 3D anatomic MRI, fractional anisotropy (FA) from diffusion tensor imaging (DTI) and resting-state functional connectivity in selected relevant large-scale networks [26–28]. Further details can be found in S1 Text.

MRI acquisition was performed using a 1.5 Tesla Signa Excite system (General Electric, Milwaukee, WI, USA) equipped with an eight-channel phased-array head coil and single-shot echo planar imaging (EPI) software was used (further details can be found in S1 Text.

Imaging data were analyzed using Statistical Parametric Mapping (SPM8) (http://www.fil.ion.ucl.ac.uk/spm, Wellcome Department of Cognitive Neurology, London, UK, 2008). Individual anatomical (cortical thickness), DTI and functional connectivity maps were included in second-level (group) analyses to map voxel-wise the correlation across-subjects between individual brain measurements and the SNP of interest. Results were considered significant with clusters of 1.032 ml (e.g., 129 voxels with a resolution of 2x2x2 mm) at a height threshold of p<0.005, which satisfied the family-wise error (FWE) rate correction of PFWE<0.05 according to recent Monte Carlo simulations [29]. Maps in figures are displayed at t>2.3.

## Results

### Descriptive results

Table 1 shows age, sex ratio and scores for the five attention outcomes of the discovery (BREATHE) and replication sample (INMA-SabVal). Within BREATHE sample, girls showed a better performance in executive attention [*t*(1493) = 4.21; P<0.001], but a worse performance in HRT [*t*(1493) = -8.39; P<0.001] and HRTSE [*t*(1493) = -5.63; P<0.001], compared to boys. Similar findings were observed within INMA-SabVal sample, in regard to sex differences for HRT [*t*(544) = 6.1; P<0.001] and HRTSE [*t*(544) = 4.6; P<0.001].

**Table 1:** Descriptive data for the variables of the study for the discovery (Breathe) and replication (INMA-SabVal) samples. Percentage is indicated for categorical variables. Mean, SD and maximum and minim are indicated for continuous variables.

	Breathe (n = 1655)	INMA-SabVal (n = 546)	Comparison
**Sex, females (%)**	789 (47.7%)	266 (48.7%)	*Chi*(1) = 0.3415; P = .599
**Age**	**9.2 (.87) (7.5/11.6)**	**7.12 (0.48) (5.36/8.55)**	***t*(1738) = 71.62; P < .001**
**Alerting**	48.3 (76.1) (-337.5/424)	54.37 (92.9) (-450/448)	*t*(826) = -1.35; P = .175
**Orienting**	36.8 (74.3) (-297/403)	33.31 (92.86) (-407.5/329)	*t*(813) = 0.78; P = .432
**Executive Attention**	**62.2 (58.0) (-182/557)**	**79.95 (84.63) (-185.5/646.5)**	***t*(740) = -4.52; P < .001**
**HRT**	**803.9 (160.4) (438/1501)**	**941.11 (186.84) (594.5/1724.5)**	***t*(855) = -15.23; P < .001**
**HRTSE**	**266.3 (87.2) (76.5/528.5)**	**317.12 (78) (97.25/507.99)**	***t*(1074) = -12.63; P < .001**

### Genome-wide association study: Discovery sample

**Fig 1:**
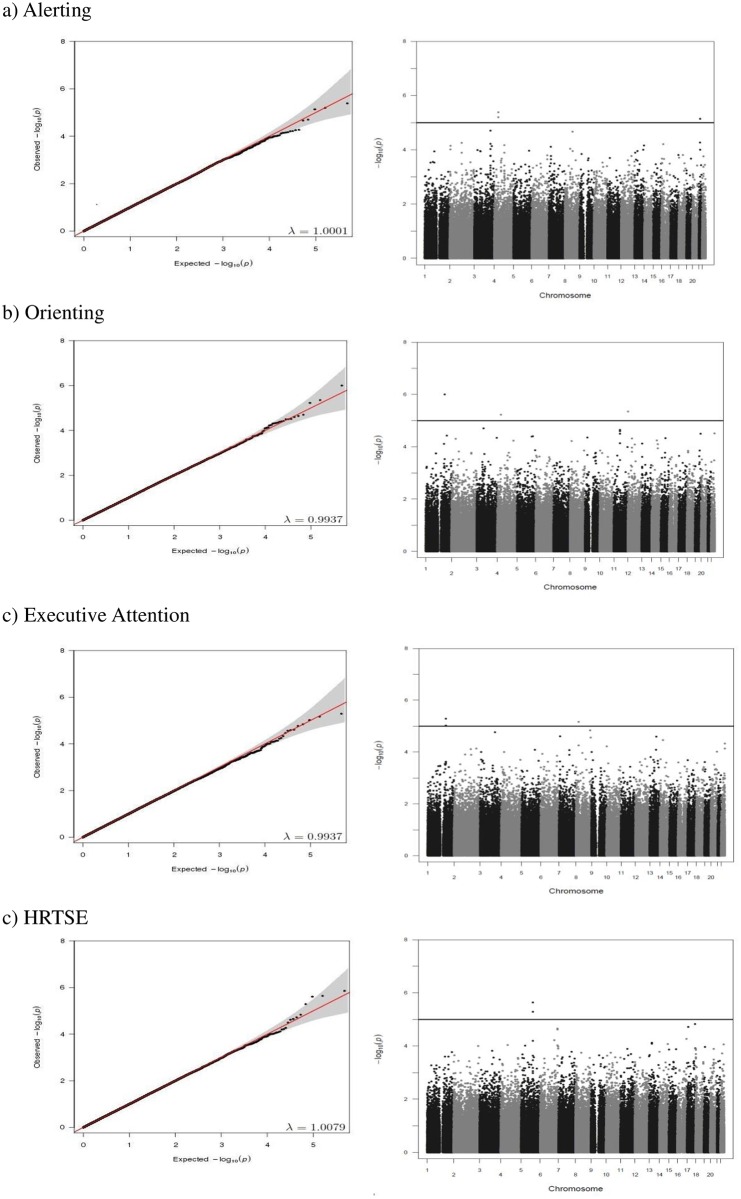
Quantile-quantile (Q-Q) plots (left side) and Manhattan plots (right side) of genome-wide association analyses for (a) alerting, (b) orienting, (c) executive attention, and (d) HRTSE attention outcomes in the discovery sample. Genomic inflation factor (λ) is included in each Q-Q plot. The blue line in the Manhattan plots indicates the suggestive level of statistical significance (P<10^−5^).

**Fig 2:**
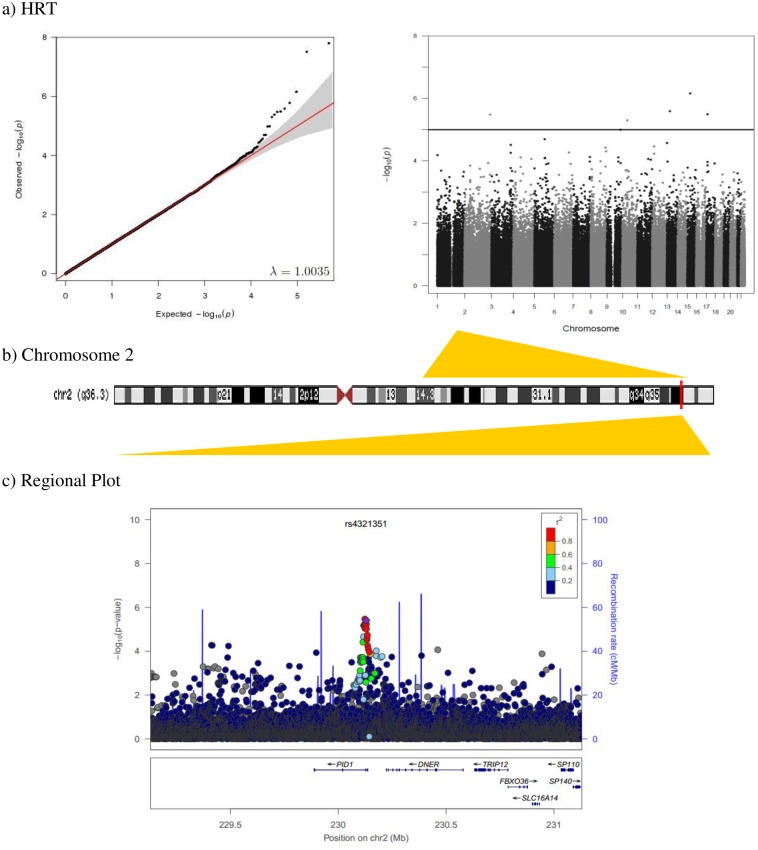
a) Quantile-quantile and Manhattan plot of genome-wide association results for HRT. The blue line indicates the suggestive level of statistical significance (p<10^−5^). b) Diagram of the chromosome 2. The red line indicates the position of the rs4321351 (230,129,493 bp). c) Regional association plot of rs4321351 located in *PID1* gene. The linkage disequilibrium (LD; r^2^) between the SNP in focus and its SNPs genotyped or imputed within 1Mb is showed in red (high LD) to blue (low LD). The recombination rate is plotted in blue according to HapMap (CEU).

Q-Q plots of the observed versus expected P-values and Manhattan plots showing the distribution of negative log-transformed P-values for every attention outcome are provided in Figs 1 and 2a. The Q-Q plots showed no departure from the expected P-values distribution. Genomic control inflation factor (λ) is included in each Q-Q plot.

No SNPs were genome-wide significant (P<10^−8^). Nevertheless, 13 loci showed suggestive evidence of association with attention outcomes (Table 2).

**Table 2:** SNPs associated with attention function outcomes at P<10^−5^ (ordered by significance).

Attention outcome	SNP	CHR	position	Allele^a^	MAF	N	β	SE	P-value	Gene	Nearest gene
Alerting	rs10015679	4	40644376	T/C	0.319	1491	-13.80	3.00	4.10 x 10^−6^	Intergenic	*RBM47*
	rs13048083	21	28286853	T/C	0.247	1491	-14.48	3.23	7.33 x 10^−6^	Intergenic	*ADAMTS5*
Orienting	rs10911457	1	183843104	T/C	0.461	1492	13.14	2.69	9.99 x 10^−7^	*RGL1*	-
	rs12579294	12	3289945	T/C	0.222	1492	-14.84	3.23	4.46 x 10^−6^	*TSPAN9*	-
	rs4629469	4	36419047	G/A	0.357	1490	12.76	2.82	5.95 x 10^−6^	Intergenic	*DTHD1*
Executive Attention	rs2207190	1	171415856	G/A	0.405	1493	-9.84	2.16	5.12 x 10^−6^	Intergenic	*PRRC2C*
	rs2320783	8	25009089	G/A	0.134	1493	-14.27	3.17	6.82 x 10^−6^	Intergenic	*DOCK5*
HRT	rs4775379	15	46682794	T/C	0.216	1484	35.87	7.23	6.98 x 10^−7^	Intergenic	*SQRDL*
	rs951738	13	45479633	G/A	0.234	1442	32.85	6.99	2.60 x 10^−6^	Intergenic	*NUFIP1*
	rs757594	17	12000632	G/A	0.233	1493	33.09	7.11	3.25 x 10^−6^	*MAP2K4*	-
	rs4321351	2	230129493	G/A	0.318	1493	-28.99	6.24	3.35 x 10^−6^	*PID1*	-
	rs6593376	10	44469148	T/C	0.215	1493	32.31	7.08	5.02 x 10^−6^	Intergenic	*LINC00841*
HRTSE	rs1560054	5	111519018	T/C	0.282	1493	-16.95	3.59	2.29 x 10^−6^	*EPB41L4A*	-

SNP, single nucleotide polymorphism; CHR, chromosome; MAF, minor allele frequency; β, regression coefficient; SE, standard error.

^a^ Effect allele/Other allele

*RBM47*, RNA binding motif protein 47; *ADAMTS5*, ADAM metallopeptidase with thrombospondin type 1 motif, 5; *RGL1*, Ral guanine nucleotide dissociation stimulator-like 1; *TSPAN9*, tetraspanin 9; *DTHD1*, death domain containing 1; *PRRC2C*, proline-rich coiled-coil 2C; *DOCK5*, dedicator of cytokinesis 5; *SQRDL*; sulfide quinone reductase-like; *NUFIP1*, nuclear fragile X mental retardation protein interacting protein 1; *MAP2K4*, dual specificity mitogen-activated protein kinase kinase 4; *PID1*, phosphotyrosine interaction domain containing 1; *LINC00841*, long intergenic non-protein coding RNA 841; *EPB41L4A*, erythrocyte membrane protein band 4.1 like 4.

The SNP with the strongest association was the rs4775379 SNP (β = 35.9, P = 6.98 x 10^−7^) associated with HRT (Table 2). The nearest gene to this intergenic SNP located on chromosome 15 is the sulfide quinone reductase-like (*SQRDL*) gene. The second most significant SNP was the rs10911457, associated with orienting (β = 13.1, P = 10.0 x 10^−7^), located on chromosome 1 in the Ral guanine nucleotide dissociation stimulator-like 1 (*RGL1*) (Table 2).

Top five most significant SNPs associated with each attention outcome can be found in S1 Table. Full summary statistics for all SNPs tested in each attention outcome can be found in S1–S5 Files.

### Genome-wide association study: Replication sample

The rs4321351 was nominally significant in the replication sample, although neither this SNP nor the others showing suggestive evidence of association with the attention outcomes remained significant after multiple testing correction (FDR<0.05).

The nominally significant SNP associated with HRT in the discovery sample (β = -29.0, P = 3.35 x 10^−6^) showed same direction of additive effect in INMA-SabVal sample (β = -27.7, P = 0.025) (Table 3). HRT scores decreased as a function of the G allele copies of the rs4321351. This SNP is located in an intronic region of the phosphotyrosine interaction domain containing 1 (*PID1*) gene. Regional association analysis within 1Mb of this loci (chr2:230129493) identified a linkage disequilibrium (LD) block of 18 SNPs (r^2^ > 0.8) yielding strong evidences of multiple association signals for HRT (Fig 2).

**Table 3:** SNPs replicated in INMA-SabVal sample.

Attention outcome	SNP	CHR	position	Allele^a^	MAF	N	β	SE	P-value	FDR
**Alerting**	rs10015679	4	40644376	T/C	0.324	545	2.95	6.08	0.628	0.891
	rs13048083	21	28286853	T/C	0.247	545	6.60	6.45	0.307	0.891
**Orienting**	rs10911457	1	183843104	C/T	0.454	546	5.10	5.65	0.367	0.891
	rs12579294	12	3289945	T/C	0.221	546	-10.09	6.98	0.148	0.858
	rs4629469	4	36419047	G/A	0.376	546	3.69	5.71	0.517	0.891
**Executive Attention**	rs2207190	1	171415856	G/A	0.430	545	-0.33	5.15	0.949	0.949
	rs2320783	8	25009089	G/A	0.146	545	-3.15	7.21	0.662	0.891
**HRT**	rs4775379	15	46682794	T/C	0.253	546	-4.46	12.74	0.726	0.891
	rs951738	13	45479633	A/G	0.241	546	5.65	12.69	0.656	0.891
	rs757594	17	12000632	A/G	0.254	546	-1.92	12.93	0.882	0.949
	rs4321351	2	230129493	G/A	0.321	546	-27.74	12.32	0.025	0.325
	rs6593376	10	44469148	T/C	0.228	546	-17.25	13.40	0.198	0.858
**HRTSE**	rs1560054	5	111519018	C/T	0.287	546	1.59	5.08	0.754	0.891

SNP, single nucleotide polymorphism; CHR, chromosome; MAF, minor allele frequency; β, regression coefficient; SE, standard error.

^a^ Effect allele/Other allele

The eQTL analysis for rs4321351 indicated that *PID1* and *DNER* genes were among the top ten most affected genes by this SNP. Moreover, exon-specific probesets in *PID1* (ID: 2602738) and *DNER* (ID: 2602778) genes were expressed in putamen (p = 0.004 and p = 0.009, respectively) according to BRAINEAC database (S2 Table).

### Gene set enrichment analysis results

Among the total of 195 functional pathways nominally associated with the attention outcomes (P<0.05), three remained significant after correcting for multiple testing (FDR<5%) (Table 4). The strongest enrichment was found for the alerting attention outcome involving the Alzheimer disease-amyloid secretase pathway (P = 9.40x10^-5^; FDR = 0.014) followed by the sex determination pathway associated with orienting (P = 6.00x10^-4^; FDR = 0.007). Also, a significant association was found for the mammalian target of rapamycin (mTOR) signalling pathway (P = 4.00x10^-4^; FDR = 0.043) for the HRT attention outcome.

**Table 4:** Gene set enrichment analysis (GSEA). Pathways significantly associated with attention outcomes after applying multiple testing correction.

				Nominal 95^th^ Percentile			
Outcome	Data Base	Gene Set	Size (n° of genes)	Expected	Observed	P-value	FDR
**Alerting**	PB	Sex determination	9	0	4	6.00 x 10^−4^	0.007
**Orienting**	RE	mTOR signalling	27	1	7	4.00 x 10^−4^	0.043
**HRT**	PA	Alzheimer disease-amyloid secretase pathway	23	1	7	9.40 x 10^−5^	0.014

RE, Reactome; PA, Panther; PB, Panther-Biological process

Top five most significant pathways associated with each attention function outcome can be found in S3 Table.

### Neuroimaging results

Although none of the SNPs were replicated, we further explored the nominally significant SNP.

**Fig 3:**
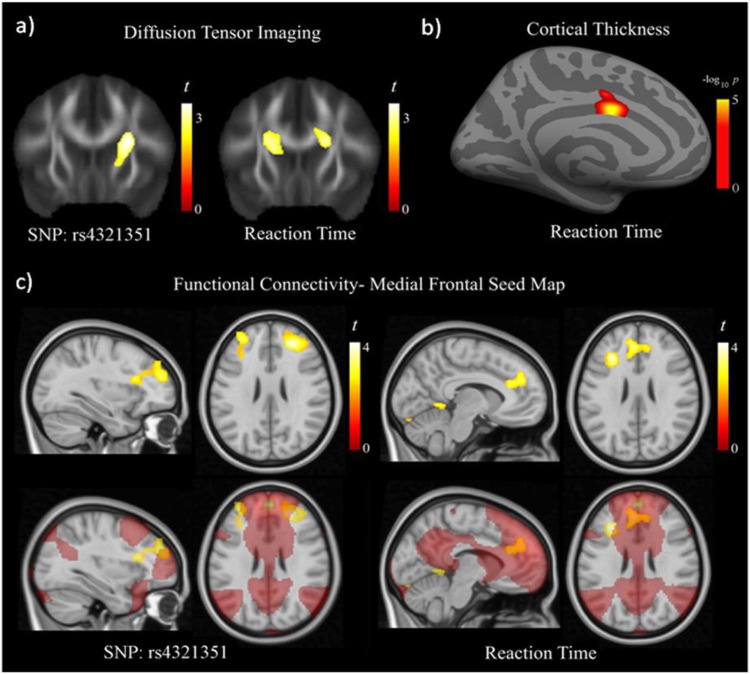
Diffusion tensor imaging results showed significantly lower fractional anisotropy (FA) in the region of the basal ganglia as a function of the G allele copies of the rs4321351 (a, left image). Lower HRT scores correlated with lower FA in this region bilaterally (a, right image) and with thinner anterior cingulate cortex (b) (cortical area of 1.2 cm2 centered at MNI, x = -6 y = 2 z = 32, Pcorrected <0.05). Functional connectivity results from the medial frontal seed map (c) showing prefrontal regions with significantly higher functional connectivity as a function of the G allele copies of the rs4321351. Lower HRT scores correlated with higher functional connectivity also in the prefrontal cortex, and in the anterior cingulate cortex. T denotes statistics t value.–log_10_ p denotes log of the probability p value. The right side corresponds to the right hemisphere in coronal and axial images.

Significant associations were detected between the rs4321351 in *PID1* gene and changes in both fractional anisotropy (FA) in DTI and functional connectivity. Specifically, carriers of the G allele of the rs4321351 presented lower FA values in the basal ganglia compared with homozygotes for the A allele (S4 Table and Fig 3a). Interestingly, lower FA in nearby white matter was associated with lower HRT (Fig 3a). Also, lower HRT was associated with thinner cortical thickness in the adjacent anterior cingulate cortex in the left hemisphere (Fig 3b). Functional results were also consistent with an effect of the SNP in frontal-basal ganglia circuits. In the medial frontal seed map, functional connectivity between the frontal medial cortex (selected seed region) and the prefrontal cortex bilaterally increased as a function of the G copies of the rs4321351 (S4 Table and Fig 3c). Increased functional connectivity in this map was associated with lower HRT involving both prefrontal cortex and anterior cingulate cortex (S4 Table and Fig 3c). Finally, in the frontal operculum seed map, carriers of the G allele presented higher functional connectivity between the frontal operculum and the basal ganglia at the putamen bilaterally (S1 Fig).

It is relevant to mention that the association between increased connectivity and lower HRT as a function of the G allele copies of the rs4321351, concerns almost selectively to the frontal-basal ganglia system; as posterior brain areas (i. e., parietal cortex), show the opposite association pattern (S4 Table).

## Discussion

To our knowledge, this is the first GWAS on attention function assessed in children from the general population. No genome-wide significant results were detected but 13 loci were identified in the suggestive range of association (P<10^−5^) in the discovery sample. One of them, the rs4321351 located in the *PID1* gene, was nominally significant in the replication sample although it did not survive multiple testing correction. This signal was further explored due to its potential relationship with the findings at the pathway level involving Alzheimer disease (AD).

The *PID1* gene increases proliferation of preadipocytes without affecting adipocytic differentiation [30]. Studies examining *PID1* has been mostly in the context of obesity and insulin resistance [30–33]. Overexpression of *PID1* in human myoblasts results in reduced insulin signaling [31] which has been pointed out as a neuroprotective agent acting mainly against apoptosis, beta amyloid toxicity, oxidative stress, and ischemia [34]. Indeed, insulin signaling has been found to be impaired in brains of patients with AD [35]; and type 2 diabetes, characterized by insulin resistance or lack of insulin, has been proposed as a risk factor for AD [36, 37]. Furthermore, *PID1* expression has been found to be significantly decreased in brains of patients with AD compared with controls [38]. These evidences suggest the involvement of *PID1* gene in neuronal processes and neurodegenerative disease. Our findings add to these evidences relating this gene with attention function during childhood.

Although the rs4321351 SNP was located within the *PID1* gene, fine-mapping results showed that the LD region was close to the Delta and Notch-like epidermal growth factor-related receptor (*DNER*) gene. Thus, we cannot discard that this SNP may be responsible for regulation of the *DNER* gene rather than *PID1* gene. DNER is a neuron-specific Notch ligand required for cerebellar development [39–41]. Also, the *DNER* gene functions as an activator of the NOTCH1 pathway which has also been related to AD and postnatal myelination and adult plasticity [42, 43]. Furthermore, copy number variations in DNER have been associated with autism spectrum disorders [44].

Additional neuroimaging analyses revealed significant associations between the genetic variant rs4321351 located at *PID1* gene and both brain structure and function with the most consistent findings involving the frontal-basal ganglia circuits. This is in accordance with models of attention consistently suggesting the interaction of cortical structures such as frontal cortex with subcortical structures such as basal ganglia to form a complex functional system implicated in sustained attention processes [45, 46]. Relevantly, individual measurement of structure and function in frontal-basal ganglia circuits showed, in turn, significant correlations with HRT where G allele carriers presented higher connectivity between these regions. The direction of the imaging results was in agreement with GWAS findings indicating that HRT scores decreased as a function of the G allele copies of the rs4321351. Furthermore, analyses based in BRAINEAC database showed that rs4321351 may act as eQTL in putamen, one of the structures comprising basal ganglia. Thus, the imaging and eQTL results reinforce the possibility that this SNP may play a role in neuronal structure and functioning related to HRT.

At the pathway level, three biological pathways were significantly associated with different attention outcomes. The sex determination pathway refers to any process that establishes and transmits the specification of sexual status of an individual organism. To our knowledge, neither the current findings nor previous research provide clear clues to link this pathway with alerting. Of note, no sex differences were detected in alerting scores. Thus, the possible role of this pathway in attention development requires replication. In contrast, the other two pathways identified, the mTOR singalling pathways and the Alzheimer disease-amyloid secretase pathway involve processes of interest for cognition which, again involve AD.

The mTOR is a ubiquitously expressed protein kinase that functions as a regulator of several cellular processes including metabolism, growth, proliferation and survival [47]. There is evidence supporting the role of mTOR signaling in synaptic plasticity and memory [48] and it has been suggested that dysregulation of mTOR signaling might be associated with neurodevelopmental, neurodegenerative and neuropsychiatric disorders [49–51]. Biological plausability for these evidences regards the modulating function of mTOR in autophagy since this signaling pathway receives inputs regarding the energetic state of the cell in order to trigger or stop the synthesis of proteins. Also, kinase mTOR is a downstream target of two other pathways, the phosphatidylinositol 3 kinase (PI3K) and kinase AKT (AKT) pathway, which together would downregulate autophagy while fostering cell growth, differentiation and survival. Therefore, activation of the PI3K/AKT/mTOR pathway would promote survival, neuronal protection, and inhibition of autophagy by mTOR activation. [50]. Interestingly, autophagy, which is partially modulated by mTOR as abovementioned, plays a critical role in multiple pathological lesions of AD, such as the formation of amyloid plaques [52] which is related to the second enriched pathway associated with attention function in our study. The AD amyloid secretase pathway refers to the role of the amyloid precursor protein (APP) in the formation of amyloid plaques in AD. However, APP is not only linked to this pathologic process, it has been suggested that APP is also involved in neurite outgrowth and synaptogenesis, neuronal protein trafficking along the axon, transmembrane signal transduction, cell adhesion, and calcium metabolism [53].

Other relevant findings include the nearest gene to the top hit SNP (rs4321351 associated with HRT at p = 6.98 x 10^−7^) the sulfide quinone reductase-like (*SQRDL*) gene, the rs10911457 (associated with orienting at p = 9.99 x 10^−7^) located in the Ral guanine nucleotide dissociation stimulator-like 1 (*RGL1)* gene, and the proline-rich coiled-coil 2C (*PRRC2C*) gene (nearest gene of the rs2207190 associated with executive attention at p = 5.12x10^-6^). The *SQRDL* is a protein-coding gene which product may function in mitochondria to catalyze the conversion of sulfide to persulfides, thereby decreasing toxic concencrations of sulfide. This gene has been related to ethylmalonic encephalopathy disease [54] and there is evidence indicating that *SQRDL* is expressed in neurons, oligodendrocytes, and endothelial cells [55]. The *RGL1* gene is involved in Ras and Ral GTPase signaling pathways as a downstream effector protein. Interestingly, it has been suggested that the functions of the Ras and Ral signaling pathways also extend into neuronal differentiation and outgrowth [56]. Furthermore, the *RGL1* gene has been associated with conduct problems in a GWAS of children with ADHD [57]. Of note, the SNP associated with conduct problems in the study of Anney and collaborators [57] (rs10797919) is in linkage disequilibrium (LD) (r^2^ = 0.60; D’ = 0.94) with the SNP within the *RGL1* gene associated with orienting in the current study (rs10911457). It might be plausible that the *RGL1* gene and its product may play a role in attention. Interestingly, the *PRRC2C* gene associated has been associated with cognitive decline in AD [58].

Of note, besides the eQTL results regarding rs4321351, the possible functionality of the genetic variants discussed above is currently unknown. To our knowledge, none of the loci were in linkage disequilibrium with any potential functional coding SNP.

It is worth mentioning that most of the relevant findings discussed above involved the HRT and HRTSE attention outcomes. Reaction Time (RT) variability is one of the most replicated deficits in ADHD [59] and previous research highlights RT as a promising cognitive target for molecular genetics investigation [60].

The current results should be interpreted considering its limitations and strengths. First, the main limitation of the study is the modest sample size which may increase type II error. Second, we examined multiple phenotypes under a massive univariate approach which may inflate type 1 error. Thus, further research and replication in larger samples are needed. That said, the strengths of the study include several aspects to overcome these limitations including, i) the use of quantitative traits and application of gene set enrichment analyses which helps increasing the power of the study, ii) the inclusion of a replication sample of a similar age and assessed with the same instrument, and iii) additional neuroimaging analyses using different techniques to get insight into the possible neural effects of the genetic variant replicated. Thus, while type II error may only be solved by increasing sample size, several genetic loci showed suggestive evidence for association with the attention outcomes analyzed. Although none of the loci was further replicated when adjusting by multiple testing, one SNP was nominally associated with the same outcome in an independent sample. Furthermore, this locus showed significant associations with different neuroimaging techniques assessing brain structure and function converging in frontal-basal ganglia connections, previously associated with attention and reaction time, as abovementioned. At pathway level, several interesting biological pathways were associated with the attention outcomes assessed underscoring proteins of interest for cognition such as mTOR and APP. Also, we used a computerized test to assess attention, the ANT, which provides homogeneous and reliable measures of attention function [17]. For these reasons, while we cannot discard that other potential genetic variants of interest would be detected in larger samples, we believe that it is unlikely that our results may be false positives since the loci, pathways and neuroimaging results obtained are likely to be biologically meaningful for attention function research.

To conclude, the current study has identified a new promising locus (rs4321351) which may be involved in attention function during childhood and is associated with brain structural and functional changes. Furthermore, to our knowledge, this is the first study suggesting that the *PID1* and the *DNER* genes, the mTOR and the amyloid precursor pathways, proposed to be involved in the pathogenesis of AD, may play a role in the development of attention function during childhood. Evidences from previous studies also suggest that cognitive functions assessed in nondemented populations may share common genetic factors with neurodegenerative disorders such as AD. AD related pathways were associated with attention outcomes in adults affected by ADHD [24]. A marginal joint effect of established AD genes was found on memory in a population-based sample of nondemented middle-aged and elderly subjects [61]. Remarkably, a recent GWAS of cognitive functions and educational attainment in UK Biobank identified genomic regions previously associated with neurodegenerative disorders and AD [62]. Thus, further research is needed to elucidate whether AD and attention function development may share common genetic and biological pathways that can be detected early in life through GWAS methodologies.

## Supporting Information

S1 Fig Functional connectivity results from the frontal operculum seed map. A bilateral portion of the putamen shows significantly higher functional connectivity with the seed region as a function of the G allele copies of the rs4321351. T denotes statistics t value. The right side corresponds to the right hemisphere in the coronal image. The sagittal image corresponds to the left hemisphere.
(DOCX)

S1 File Full summary statistics for all SNPs tested in alerting. (GZ)

S2 File Full summary statistics for all SNPs tested in orienting. (GZ)

S3 File Full summary statistics for all SNPs tested in executive function. (GZ)

S4 File Full summary statistics for all SNPs tested in HTR. (GZ)

S5 File Full summary statistics for all SNPs tested in HTRSE. (GZ)

S1 Table Five top most significant associated SNPs with attention function outcomes (ordered by significance). (DOCX)

S2 Table Top ten most affected genes by rs4321351 and relative p-values according to BRAINEAC database. (DOCX)

S3 Table Gene set enrichment analysis (GSEA) ordered by P-value. Five top most significant associated pathways with attention outcomes.
(DOCX)

S4 Table Neuroimaging results showing the association between the rs4321351 SNP (reference category: G allele homozygotes) and fractional anisotropy (FA) and functional connectivity. (DOCX)

S1 Text MRI acquisition and image preprocessing details. (DOCX)
