# Supplementary material for: A Genome-Wide Association Study of Attention Function in a Population-Based Sample of Children
Source: PLoS One. 2016 Sep 22;11(9):e0163048. doi: 10.1371/journal.pone.0163048 (PMC5033492; doi:10.1371/journal.pone.0163048)
Supplement: S1 Fig — A bilateral portion of the putamen shows significantly higher functional connectivity with the seed region as a function of the G allele copies of the rs4321351. T denotes statistics t value. The right side corresponds to the right hemisphere in the coronal image. The sagittal image corresponds to the left hemisphere. (DOCX) [file pone.0163048.s001.docx]

**
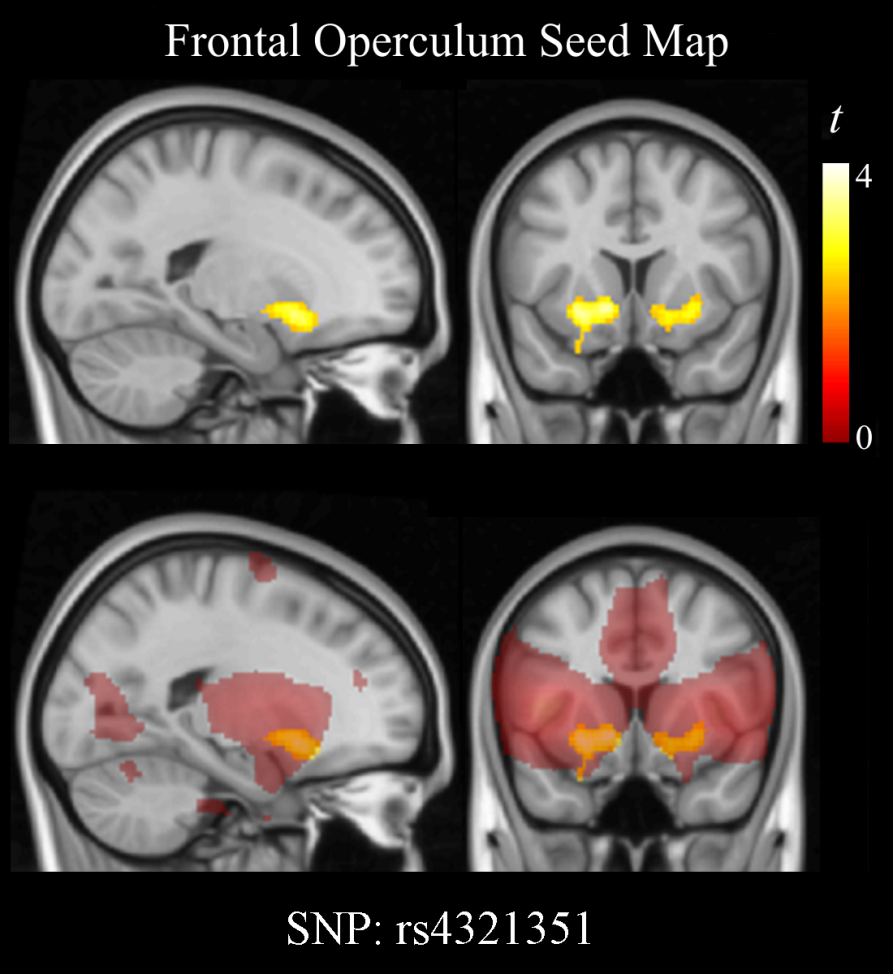
S1 Figure.** **Functional connectivity results from the frontal operculum seed map.** A bilateral portion of the putamen shows significantly higher functional connectivity with the seed region as a function of the G allele copies of the rs4321351. T denotes statistics t value. The right side corresponds to the right hemisphere in the coronal image. The sagittal image corresponds to the left hemisphere.
