# Supplementary material for: A Genome-Wide Association Study of Attention Function in a Population-Based Sample of Children
Source: PLoS One. 2016 Sep 22;11(9):e0163048. doi: 10.1371/journal.pone.0163048 (PMC5033492; doi:10.1371/journal.pone.0163048)
Supplement: S1 Table — (DOCX) [file pone.0163048.s007.docx]

| **S1 Table.** **Five top most significant associated SNPs with attention function outcomes (ordered by significance).** | | | | | | | | | | | |  |
| --- | --- | --- | --- | --- | --- | --- | --- | --- | --- | --- | --- | --- |
| **Attention outcome** | **SNP** | **CHR** | **position** | **Allele^a^** | **MAF** | **N** | **β** | **SE** | **P-value** | **Gene** | **Nearest gene** | **Rank** |
| **Alerting** | rs10015679 | 4 | 40644376 | T/C | 0.319 | 1491 | -13.80 | 3.00 | 4.10 x 10^-6^ | Intergenic | *RBM47* | *1* |
|  | rs13048083 | 21 | 28286853 | T/C | 0.247 | 1491 | -14.48 | 3.23 | 7.33 x 10^-6^ | Intergenic | *ADAMTS5* | *2* |
|  | rs2916163 | 8 | 75798282 | T/C | 0.360 | 1490 | 12.201 | 2.87 | 2.17 x 10^-5^ | Intergenic | *AKA024242* | *3* |
|  | rs12482125 | 21 | 26783415 | G/A | 0.193 | 1491 | 14.24 | 3.52 | 5.34 x 10^-5^ | *LINC00158* | *-* | *4* |
|  | rs9821185 | 3 | 161226185 | T/C | 0.375 | 1485 | 11.37 | 2.84 | 6.06 x 10^-5^ | Intergenic | *OTOL1* | *5* |
| **Orienting** | rs10911457 | 1 | 183843104 | T/C | 0.461 | 1492 | 13.14 | 2.69 | 9.99 x 10^-7^ | *RGL1* | - | *1* |
|  | rs12579294 | 12 | 3289945 | T/C | 0.222 | 1492 | -14.84 | 3.23 | 4.46 x 10^-6^ | *TSPAN9* | - | *2* |
|  | rs4629469 | 4 | 36419047 | G/A | 0.357 | 1490 | 12.76 | 2.82 | 5.95 x 10^-6^ | Intergenic | *DTHD1* | *3* |
|  | rs6762527 | 3 | 66003745 | T/C | 0.373 | 1491 | 12.22 | 2.86 | 2.00 x 10 ^-5^ | *MAGI1* | *-* | *4* |
|  | rs482386 | 11 | 60400154 | T/C | 0.361 | 1492 | -11.86 | 2.80 | 2.29 x 10 ^-5^ | *LINC00301* | *-* | *5* |
| **Executive Attention** | rs2207190 | 1 | 171415856 | G/A | 0.405 | 1493 | -9.84 | 2.16 | 5.12 x 10^-6^ | Intergenic | *PRRC2C* | *1* |
|  | rs2320783 | 8 | 25009089 | G/A | 0.134 | 1493 | -14.27 | 3.17 | 6.82 x 10^-6^ | Intergenic | *DOCK5* | *2* |
|  | rs1901090 | 8 | 133497127 | G/A | 0.330 | 1493 | -9.84 | 2.27 | 1.44 x 10 ^-5^ | Intergenic | *KCNQ3* | *3* |
|  | rs4368461 | 3 | 139832777 | T/C | 0.266 | 1493 | 10.50 | 2.44 | 1.70 x 10 ^-5^ | *CLSTN2* |  | *4* |
|  | rs12539924 | 7 | 10343038 | G/A | 0.345 | 1493 | -9.23 | 2.19 | 2.47 x 10 ^-5^ | Intergenic | *NDUFA4* | *5* |
| **HRT** | rs4775379 | 15 | 46682794 | T/C | 0.216 | 1484 | -35.87 | 7.23 | 6.98 x 10^-7^ | Intergenic | *SQRDL* | *1* |
|  | rs951738 | 13 | 45479633 | G/A | 0.234 | 1442 | 32.85 | 6.99 | 2.60 x 10^-6^ | Intergenic | *NUFIP1* | *2* |
|  | rs757594 | 17 | 12000632 | G/A | 0.233 | 1493 | 33.09 | 7.11 | 3.25 x 10^-6^ | *MAP2K4* | - | *3* |
|  | rs4321351 | 2 | 230129493 | G/A | 0.318 | 1493 | -28.99 | 6.24 | 3.35 x 10^-6^ | *PID1* | - | *4* |
|  | rs6593376 | 10 | 44469148 | T/C | 0.215 | 1493 | 32.31 | 7.08 | 5.02 x 10^-6^ | Intergenic | *LINC00841* | *5* |
| **HRTSE** | rs1560054 | 5 | 111519018 | T/C | 0.282 | 1493 | -16.95 | 3.59 | 2.29 x 10^-6^ | *EPB41L4A* | - | *1* |
|  | rs757594 | 17 | 12000632 | G/A | 0.233 | 1493 | 16.51 | 3.86 | 1.91 x 10 ^-5^ | *MAP2K4* | - | *2* |
|  | rs1084656 | 6 | 161181292 | C/A | 0.456 | 1493 | -13.63 | 3.21 | 2.24 x 10 ^-5^ | Intergenic | *PLG* | *3* |
|  | rs13336802 | 16 | 84412028 | G/A | 0.435 | 1493 | 12.92 | 3.20 | 5.39 x 10 ^-5^ | *ATP2C2* | - | *4* |
|  | rs928501 | 6 | 132275437 | T/G | 0.260 | 1493 | -14.98 | 3.73 | 6.09 x 10 ^-5^ | Intergenic | *CTGF* | *5* |
| SNP, single nucleotide polymorphism; CHR, chromosome; MAF, minor allele frequency; β, regression coefficient; SE, standard error.  ^a^ Effect allele/Other allele  *RBM47*, RNA binding motif protein 47; *ADAMTS5*, ADAM metallopeptidase with thrombospondin type 1 motif, 5; *RGL1*, Ral guanine nucleotide dissociation stimulator-like 1; *TSPAN9*, tetraspanin 9; *DTHD1*, death domain containing 1; *MAGI1*, membrane associated guanylate kinase, WW and PDZ domain containing 1;  *LINC00301,* long intergenic non-protein coding RNA 301; *PRRC2C*, proline-rich coiled-coil 2C; *DOCK5*, dedicator of cytokinesis 5; *KCNQ3,* potassium voltage-gated channel, KQT-like subfamily, member 3; *CLSTN2*, calsyntenin 2; *NDUFA4,* mitochondrial complex associated; *SQRDL*; sulfide quinone reductase-like; *NUFIP1*, nuclear fragile X mental retardation protein interacting protein 1; *MAP2K4*, dual specificity mitogen-activated protein kinase kinase 4; *PID1*, phosphotyrosine interaction domain containing 1; *LINC00841*, long intergenic non-protein coding RNA 841; *EPB41L4A*, erythrocyte membrane protein band 4.1 like 4; *PLG*, plasminogen;  *ATP2C2,*  ATPase, Ca++ transporting, type 2C, member 2*; CTGF,* connective tissue growth factor. | | | | | | | | | | | | |
