# Supplementary material for: A Genome-Wide Association Study of Attention Function in a Population-Based Sample of Children
Source: PLoS One. 2016 Sep 22;11(9):e0163048. doi: 10.1371/journal.pone.0163048 (PMC5033492; doi:10.1371/journal.pone.0163048)
Supplement: S2 Table — (DOCX) [file pone.0163048.s008.docx]

| **S2 Table. Top ten most affected genes by rs4321351 and relative p-values according to BRAINEAC database.** | | | | | | | | | | | | | | | | |
| --- | --- | --- | --- | --- | --- | --- | --- | --- | --- | --- | --- | --- | --- | --- | --- | --- |
| **Gene Symbol** | **Marker** | **rsid** | **exprID** | **Start** | **Stop** | **aveALL** | **CRBL** | **FCTX** | **HIPP** | **MEDU** | **OCTX** | **PUTM** | **SNIG** | **TCTX** | **THAL** | **WHMT** |
| TRIP12,  LOC100131015 | chr2:  230129493 | rs4321351 | 2602921 | 230630801 | 230786705 | 8.5x10^-3^ | 5.9e-04 | 9.3e-02 | 5.2e-01 | 6.1e-01 | 6.5e-01 | 7.1e-02 | 4.6e-01 | 8.5e-03 | 4.8e-01 | 7.5e-01 |
| SP110 | chr2:  230129493 | rs4321351 | 2603078 | 231032015 | 231089568 | 3.8e-01 | 7.2e-02 | 7.9e-01 | 1.0e-03 | 6.9e-01 | 1.6e-01 | 4.9e-01 | 8.6e-01 | 7.5e-01 | 1.5e-01 | 4.1e-01 |
| TRIP12,  LOC100131015 | chr2:  230129493 | rs4321351 | 2602957 | 230630801 | 230786705 | 8.5e-02 | 3.2e-01 | 7.6e-01 | 1.2e-01 | 5.7e-01 | 9.0e-01 | 3.4e-03 | 6.8e-01 | 4.4e-02 | 8.1e-01 | 3.7e-01 |
| PID1 | chr2:  230129493 | rs4321351 | 2602738 | 229715085 | 230137889 | 8.9e-02 | 5.7e-01 | 5.2e-01 | 1.8e-01 | 4.0e-01 | 4.3e-01 | 4.4e-03 | 9.0e-01 | 5.3e-02 | 8.8e-02 | 3.3e-01 |
| SP110 | chr2  :230129493 | rs4321351 | 2603063 | 231032015 | 231089568 | 2.8e-01 | 7.4e-01 | 7.4e-01 | 6.9e-01 | 6.1e-01 | 8.4e-01 | 6.9e-01 | 8.3e-01 | 2.6e-01 | 2.5e-01 | 4.7e-03 |
| TRIP12,  LOC100131015 | chr2:  230129493 | rs4321351 | 2602954 | 230630801 | 230786705 | 2.9e-01 | 7.0e-01 | 3.0e-01 | 5.4e-01 | 5.8e-01 | 4.6e-01 | 1.3e-02 | 7.9e-01 | 4.8e-03 | 8.4e-01 | 4.6e-01 |
| SP140,  SP140L | chr2:  230129493 | rs4321351 | 2531259 | 231090446 | 231177928 | 1.3e-01 | 9.3e-01 | 6.2e-03 | 2.0e-01 | 9.6e-01 | 5.6e-01 | 4.8e-02 | 6.4e-01 | 7.5e-01 | 5.8e-02 | 9.5e-01 |
| DNER | chr2:  230129493 | rs4321351 | 2602778 | 230110640 | 230582911 | 3.5e-01 | 1.5e-01 | 8.5e-01 | 3.0e-01 | 8.5e-01 | 9.2e-02 | 8.5e-03 | 8.7e-01 | 6.8e-01 | 5.4e-01 | 7.2e-01 |
| SP140,  SP140L | chr2:  230129493 | rs4321351 | 2531239 | 231090446 | 231177928 | 1.8e-01 | 8.5e-01 | 9.8e-02 | 9.5e-01 | 3.2e-01 | 2.5e-01 | 1.0e-02 | 3.1e-01 | 3.8e-02 | 2.1e-01 | 2.1e-02 |
| SLC16A14 | chr2  :230129493 | rs4321351 | 2603002 | 230886046 | 230943520 | 1.1e-01 | 1.1e-01 | 8.9e-01 | 1.7e-01 | 8.7e-01 | 6.2e-01 | 5.1e-01 | 9.0e-01 | 1.1e-02 | 4.1e-01 | 1.6e-01 |
| GeneSymbol, gene name; Marker, position of the SNP inserted; rsid, SNP ID; exprID, exon-specific probeset affected by the SNP; aveAll, expression profile across all the tissues; SNIG, substantia nigra; PUTM, putamen (at the level of the anterior commissure); MEDU, the inferior olivary nucleus (sub-dissected from the medulla); THAL, thalamus (at the level of the lateral geniculate nucleus); OCTX, occipital cortex; HIPP, hippocampus; FCTX, frontal cortex; TCTX, temporal cortex; WHMT, intralobular white matter; CRBL, cerebellar cortex. | | | | | | | | | | | | | | | | |
