# Supplementary material for: A Genome-Wide Association Study of Attention Function in a Population-Based Sample of Children
Source: PLoS One. 2016 Sep 22;11(9):e0163048. doi: 10.1371/journal.pone.0163048 (PMC5033492; doi:10.1371/journal.pone.0163048)
Supplement: S3 Table — Five top most significant associated pathways with attention outcomes. (DOCX) [file pone.0163048.s009.docx]

| **S3 Table. Gene set enrichment analysis (GSEA) ordered by P-value.** Five top most significant associated pathways with attention outcomes. | | | | | | | | |
| --- | --- | --- | --- | --- | --- | --- | --- | --- |
|  | | | | **Nominal 95^th^ Percentile** | |  |  |  |
| **Outcome** | **Data Base** | **Gene Set** | **Size (Nº of genes)** | **Expected** | **Observed** | **P-value** | **FDR 95^th^** | **Rank** |
| **Alerting** | PB | Sex determination | 9 | 0 | 4 | 6.00 x 10^-4^ | 0.007 | *1* |
|  | RE | Cell junction organization | 77 | 4 | 11 | 1.20 x 10^-3^ | 0.200 | *2* |
|  | RE | MTORC1 mediated signalling | 11 | 1 | 4 | 1.70 x 10^-3^ | 0.115 | *3* |
|  | PB | mRNA transcription regulation | 1047 | 52 | 73 | 2.20 x 10^-3^ | 0.321 | *4* |
|  | RE | Amino acid synthesis and conversion | 12 | 1 | 4 | 2.60 x 10^-3^ | 0.108 | *5* |
| **Orienting** | RE | MTOR signalling | 27 | 1 | 7 | 4.00 x 10^-4^ | 0.042 | *1* |
|  | RE | Gap junction degradation | 10 | 1 | 4 | 9.00 x 10^-4^ | 0.625 | *2* |
|  | RE | MTORC1 mediated signalling | 11 | 1 | 4 | 1.40 x 10^-3^ | 0.516 | *3* |
|  | PB | Receptor mediated endocytosis | 95 | 5 | 12 | 1.70 x 10^-3^ | 0.492 | *4* |
|  | PM | Vesicle coat protein | 40 | 2 | 7 | 3.20 x 10^-3^ | 0.475 | *5* |
| **Executive Attention** | PM | Membrane traffic regulatory protein | 91 | 5 | 13 | 9.00 x 10^-4^ | 0.207 | *1* |
|  | PM | Non-motor actin binding protein | 136 | 7 | 15 | 2.70 x 10^-3^ | 0.284 | *2* |
|  | RE | Phosphorylation of CD3 and zeta chains | 7 | 0 | 3 | 3.70 x 10^-3^ | 0.179 | *3* |
|  | PM | Select regulatory molecule | 68 | 3 | 9 | 4.80 x 10^-3^ | 0.261 | *4* |
|  | IN | T cell receptor signaling | 33 | 2 | 6 | 5.30 x 10^-3^ | 0.146 | *5* |
| **HRT** | PA | Alzheimer disease-amyloid secretase pathway | 23 | 1 | 7 | 9.40 x 10^-5^ | 0.014 | *1* |
|  | PB | Other signal transduction | 52 | 3 | 9 | 5.00 x 10^-4^ | 0.146 | *2* |
|  | RE | Regulated proteolysis of P75NTR | 11 | 1 | 4 | 1.40 x 10^-3^ | 0.142 | *3* |
|  | PM | Double-stranded DNA binding protein | 20 | 1 | 5 | 2.60 x 10^-3^ | 0.174 | *4* |
|  | KE | Other glycan degradation | 15 | 1 | 4 | 4.90 x 10^-3^ | 0.176 | *5* |
| **HRTSE** | PA | TCA cycle | 13 | 1 | 4 | 3.00 x 10^-3^ | 0.120 | *1* |
|  | RE | Formation of the ternary complex and subsequently the 43S complex | 42 | 2 | 7 | 4.30 x 10^-3^ | 0.326 | *2* |
|  | RE | Diabetes pathways | 342 | 17 | 28 | 6.70 x 10^-3^ | 0.640 | *3* |
|  | RE | Regulation of insulin like growth factor activity by insulin like growth factor binding proteins | 17 | 1 | 4 | 7.30 x 10^-3^ | 0.429 | *4* |
|  | PB | Other nucleoside, nucleotide and nucleic acid metabolism | 27 | 1 | 5 | 9.20 x 10^-3^ | 0.888 | *5* |
| RE, Reactome; PA, Panther; PB, Panther-Biological process; PM, Panther-Molecular function; KE, KEGG; IN, Ingenuity. | | | | | | | | |
