# Supplementary material for: A Genome-Wide Association Study of Attention Function in a Population-Based Sample of Children
Source: PLoS One. 2016 Sep 22;11(9):e0163048. doi: 10.1371/journal.pone.0163048 (PMC5033492; doi:10.1371/journal.pone.0163048)
Supplement: S4 Table — (DOCX) [file pone.0163048.s010.docx]

| **S4 Table. Neuroimaging results showing the association between the rs4321351 SNP (reference category: G allele homozygotes) and fractional anisotropy (FA) and functional connectivity.** | | | |
| --- | --- | --- | --- |
| **Diffusion Tensor Imaging (DTI)- FA** | | | |
|  | *Cluster size, ml* | *x y z* | *t* |
| **rs4321351** |  |  |  |
| R Basal Ganglia region- negative correlation | 1.3 | 25 18 20 | 3.6 |
| **Reaction time** |  |  |  |
| R Basal Ganglia region- positive correlation | 3.6 | 19 15 20 | 4.1 |
| L Basal Ganglia region- positive correlation | 4.2 | -17 10 19 | 3.8 |
| **Functional MRI- Functional Connectivity** | | | |
| ***Medial Frontal Seed Map*** | | | |
| **rs4321351** |  |  |  |
| L Prefrontal Cortex - positive correlation | 1.7 | -30 48 26 | 3.4 |
| R Prefrontal Cortex - positive correlation | 3.6 | 26 42 28 | 4.3 |
| **Reaction time** |  |  |  |
| L Prefrontal Cortex - negative correlation | 1.9 | -30 26 28 | 4.3 |
| Medial Frontal /Anterior Cingulate Cortex - negative correlation | 2.8 | -4 40 26 | 3.5 |
| Visual cortex - negative correlation | 1.1 | 2 -72 -6 | 3.7 |
| ***Posterior Cingulate Cortex Seed Map*** | | | |
| **rs4321351** |  |  |  |
| R Parietal Cortex - negative correlation | 5.7 | 28 -52 40 | 4.2 |
| Posterior Cingulate Cortex - negative correlation | 2.1 | -8 30 46 | 4.0 |
| **Reaction time** |  |  |  |
| R Parietal Cortex - positive correlation | 1.4 | -40 -86 14 | 3.6 |
| L Parahippocampal gyrus - negative correlation | 4.2 | -32 -40 -8 | 4.7 |
| R Parahippocampal gyrus - negative correlation | 3.4 | 24 -34 -8 | 3.5 |
| ***Dorsal Frontal Seed Map*** | | | |
| **rs4321351** |  |  |  |
| R Thalamus - positive correlation | 1.9 | 18 -14 10 | 3.3 |
| **Reaction time** |  |  |  |
| Posterior CingulateCortex - positive correlation | 2.2 | -10 -34 40 | 4.1 |
| Precuneus - positive correlation | 7.8 | 6 -64 48 | 3.4 |
| R Precentral gyrus - positive correlation | 1.5 | 28 -18 66 | 3.5 |
| ***Supplementary Motor Area Seed Map*** | | | |
| **rs4321351** |  |  |  |
| R Prefrontal Cortex - positive correlation | 5.1 | 42 44 4 | 3.9 |
| L Prefrontal Cortex - positive correlation | 3.0 | -40 36 16 | 3.5 |
| R Frontal Operculum/Anterior Insula - positive correlation | 4.6 | 54 18 -6 | 3.7 |
| **Reaction time** |  |  |  |
| R Premotor Cortex- negative correlation | 2.2 | 38 -4 54 | 3.6 |
| ***Frontal Operculum Seed Map*** | | | |
| **rs4321351** |  |  |  |
| R Putamen - positive correlation | 1.6 | 28 8 -8 | 3.4 |
| L Putamen - positive correlation | 5.2 | -28 6 -8 | 4.3 |
| R Parahippocampal gyrus - positive correlation | 1.3 | 30 -16 -22 | 3.1 |
| L Parahippocampal gyrus - positive correlation | 1.7 | -28 -20 -24 | 3.7 |
| **Reaction time** |  |  |  |
| (No significant results were detected) |  |  |  |
| R, right. L, left. | | | |
