# Supplementary material for: A Genome-Wide Association Study of Attention Function in a Population-Based Sample of Children
Source: PLoS One. 2016 Sep 22;11(9):e0163048. doi: 10.1371/journal.pone.0163048 (PMC5033492; doi:10.1371/journal.pone.0163048)
Supplement: S1 Text — (DOCX) [file pone.0163048.s011.docx]

**S1 Text. MRI acquisition and image preprocessing details.**

High-resolution 3D anatomical images were obtained using an axial T1-weighted three dimensional fast spoiled gradient inversion recovery-prepared sequence. A total of 134 contiguous slices were acquired with repetition time 11.9 ms; echo time 4.2 ms; flip angle 15o; field of view 30 cm; 256 x 256 pixel matrix; slice thickness 1.2 mm. All the anatomical images were visually inspected and subjects with poor quality images were discarded. Cortical thickness measurements across the whole cortex were obtained using FreeSurfer tools (http://surfer.nmr.mgh.harvard.edu/). Processing steps included removal of non-brain tissue, segmentation of the subcortical white matter and deep gray matter volumetric structures, tessellation of the gray and white matter boundary, registration to a spherical atlas which is based on individual cortical folding patterns to match cortical geometry across subjects and creation of a variety of surface based data. Cortical thickness is calculated as the closest distance from the gray/white boundary to the gray/CSF boundary at each vertex on the tessellated surface (1).

Diffusion tensor imaging (DTI) was obtained using spin-echo single-shot echo-planar sequences of 25 directions with a B-factor of 1000 s/mm2. Twenty-six slices were acquired with repetition time 8300 ms; echo time 94 ms; thickness 5 mm, no gap; pulse angle 90°; field of view 26 cm; 128 x 128 acquisition matrix reconstructed into a 256 x 256 matrix. DTI was processed using Functional MRI of the Brain (FMRIB) Software Library 5.0 (FSL), developed by the Analysis Group at the Oxford Centre for FMRIB (2). Diffusion weighted images were corrected for motion and eddy current distortions (“Eddy Current Correction” option in the FMRIB Diffusion Toolbox [FDT] version 2.0 in FSL), and a whole-brain mask was applied using the FSL Brain Extracting Tool. A further rigorous image quality control was carried out to identify potential residual effects of head motion, which involved the visual inspection of each DTI slice for all 25 DTI volumes in all participants. Volumes with slices with signal loss (greater than ~ 10%) or residual artifacts were identified by an expert researcher. DTI full examinations showing one or more degraded images in more than 5 volumes were discarded. Fractional anisotropy (FA) maps were estimated using FDT in FSL after local fitting of the diffusion tensor model at each voxel (“dtifit”). Next, diffusion data were processed using Tract- Based Spatial Statistics18. Each FA data set was re-sliced to a 1mm × 1mm × 1 mm anatomical resolution and normalized to standard MNI space via the FMRIB58_FA template using the FMRIB’s Non-linear Registration Tool.

The functional MRI sequence consisted of gradient recalled acquisition in the steady state with repetition time 2000 ms; echo time 50 ms; pulse angle 90^o^; field of view 24 cm; 64 x 64-pixel matrix; slice thickness 4 mm (inter-slice gap, 1.5 mm). Twenty-two interleaved slices were prescribed parallel to the anterior-posterior commissure line covering the whole-brain. A 6-min continuous resting-state scan was acquired for each participant. Children were instructed to relax, stay awake and lie still without moving, while keeping their eyes closed throughout. This scan generated 180 whole-brain EPI volumes. The first four (additional) images in each run were discarded to allow magnetization to reach equilibrium. Preprocessing was carried out using SPM8 and involved motion correction, spatial normalization and smoothing using a Gaussian filter (full-width half-maximum, 8 mm). Data were normalized to the standard SPM-EPI template and re-sliced to 2 mm isotropic resolution in MNI space via the FMRIB58_FA template using the FMRIB’s Non-linear Registration Tool. Functional connectivity maps were generated using procedures detailed in previous reports (3, 4) and coordinates taken from a classical study (5), converted to MNI and located at the medial frontal cortex [x=1, y=54, z=26], posterior cingulate cortex [x=-2, y=-38, z=38], dorsal frontal cortex [x=28, y=-10, z=58], supplementary motor area [x=-2, y=-2, z=55] and frontal operculum [x=-45, y=5 z=9]. The brain networks identified in the resulting maps have proposed to characterize the functional organization of the brain at its largest scale (5) and may serve as global functional assessment in the present study.

The following procedures were adopted to control for potential head motion effects: (i) Conventional SPM time-series alignment to the first image volume in each subject. (ii) Exclusion of children with large head motion (boxplot-defined outliers) (6). (iii) Both motion-related regressors and estimates of global brain signal fluctuations were included as confounding variables in first-level (single-subject) analyses. (iv) Within-subject, censoring-based MRI signal artifact removal (7) (scrubbing) was used to discard motion-affected volumes. For each subject, inter-frame motion measurements (6) served as an index of data quality to flag volumes of suspect quality across the run. At points with inter-frame motion > 0.2 mm, that corresponding volume, the immediately preceding and the succeeding two volumes were discarded. Using this procedure, a mean ± SD of 11.2 (6.2 %) ± 13.8 volumes out of 180 fMRI resting-state sequence volumes were removed. (v) Potential motion effects were further removed using a summary measurement for each participant (mean inter-frame motion across the fMRI run) as a regressor in the second-level (group) analyses in SPM (6).

**References**

1. Fischl B, Sereno MI, Dale AM. Cortical surface-based analysis. II: Inflation, flattening, and a surface-based coordinate system. Neuroimage. 1999 Feb;9(2):195-207.

2. Smith SM, Jenkinson M, Woolrich MW, Beckmann CF, Behrens TE, Johansen-Berg H, et al. Advances in functional and structural MR image analysis and implementation as FSL. Neuroimage. 2004;23 Suppl 1:S208-19.

3. Harrison BJ, Pujol J, Cardoner N, Deus J, Alonso P, Lopez-Sola M, et al. Brain corticostriatal systems and the major clinical symptom dimensions of obsessive-compulsive disorder. Biol Psychiatry. 2013 Feb 15;73(4):321-8.

4. Pujol J, Del Hoyo L, Blanco-Hinojo L, de Sola S, Macia D, Martinez-Vilavella G, et al. Anomalous brain functional connectivity contributing to poor adaptive behavior in Down syndrome. Cortex. 2014 Mar;64:148-56.

5. Fox MD, Snyder AZ, Vincent JL, Corbetta M, Van Essen DC, Raichle ME. The human brain is intrinsically organized into dynamic, anticorrelated functional networks. Proc Natl Acad Sci U S A. 2005 Jul 5;102(27):9673-8.

6. Pujol J, Macia D, Blanco-Hinojo L, Martinez-Vilavella G, Sunyer J, de la Torre R, et al. Does motion-related brain functional connectivity reflect both artifacts and genuine neural activity? Neuroimage. 2014 Nov 1;101:87-95.

7. Power JD, Mitra A, Laumann TO, Snyder AZ, Schlaggar BL, Petersen SE. Methods to detect, characterize, and remove motion artifact in resting state fMRI. Neuroimage. 2014 Jan 1;84:320-41.
